# Automated surveillance of non-ventilator-associated hospital-acquired pneumonia (nvHAP): a systematic literature review

**DOI:** 10.1186/s13756-024-01375-8

**Published:** 2024-03-06

**Authors:** Aline Wolfensberger, Alexandra U. Scherrer, Hugo Sax

**Affiliations:** 1 Department of Infectious Diseases and Hospital Epidemiology, University Hospital Zurich, University of Zurich, Rämistrasse 100, 8091 Zurich, Switzerland; 2 Institute for Implementation Science in Healthcare, University of Zurich, Zurich, Switzerland; 3 Department of Infectious Diseases, Bern University Hospital and University of Bern, Bern, Switzerland

**Keywords:** Non-ventilator-associated hospital-acquired pneumonia, Healthcare-associated pneumonia, Surveillance, Electronic surveillance, Automated surveillance, Systematic literature review, Review

## Abstract

**Background:**

Hospital-acquired pneumonia (HAP) and its specific subset, non-ventilator hospital-acquired pneumonia (nvHAP) are significant contributors to patient morbidity and mortality. Automated surveillance systems for these healthcare-associated infections have emerged as a potentially beneficial replacement for manual surveillance. This systematic review aims to synthesise the existing literature on the characteristics and performance of automated nvHAP and HAP surveillance systems.

**Methods:**

We conducted a systematic search of publications describing automated surveillance of nvHAP and HAP. Our inclusion criteria covered articles that described fully and semi-automated systems without limitations on patient demographics or healthcare settings. We detailed the algorithms in each study and reported the performance characteristics of automated systems that were validated against specific reference methods. Two published metrics were employed to assess the quality of the included studies.

**Results:**

Our review identified 12 eligible studies that collectively describe 24 distinct candidate definitions, 23 for fully automated systems and one for a semi-automated system. These systems were employed exclusively in high-income countries and the majority were published after 2018. The algorithms commonly included radiology, leukocyte counts, temperature, antibiotic administration, and microbiology results. Validated surveillance systems' performance varied, with sensitivities for fully automated systems ranging from 40 to 99%, specificities from 58 and 98%, and positive predictive values from 8 to 71%. Validation was often carried out on small, pre-selected patient populations.

**Conclusions:**

Recent years have seen a steep increase in publications on automated surveillance systems for nvHAP and HAP, which increase efficiency and reduce manual workload. However, the performance of fully automated surveillance remains moderate when compared to manual surveillance. The considerable heterogeneity in candidate surveillance definitions and reference standards, as well as validation on small or pre-selected samples, limits the generalisability of the findings. Further research, involving larger and broader patient populations is required to better understand the performance and applicability of automated nvHAP surveillance.

**Supplementary Information:**

The online version contains supplementary material available at 10.1186/s13756-024-01375-8.

## Background

Non-ventilator-associated hospital-acquired pneumonia (nvHAP) is a specific subset of hospital-acquired pneumonia (HAP) that affects patients without an invasive respiratory assist device, thereby differentiating it from ventilator-associated pneumonia (VAP) [1]. Despite being one of the most common healthcare-associated infections (HAIs) [2–4], and having considerable implications for patient morbidity, mortality, and healthcare expenditure [5], and noteworthy contribution to heightened antibiotic use, nvHAP has long been overlooked by the infection prevention and control (IPC) community [6, 7]. Recently, the importance and unique risk factors of nvHAP have led to the inclusion of nvHAP in internationally recognised IPC guidelines [8], and research into interventions to mitigate nvHAP has been gaining momentum over the past five years [9–12].

Surveillance is widely recognised as a fundamental aspect of infection prevention and control (IPC), instrumental in detecting outbreaks, shaping preventative initiatives, and assessing the efficacy of interventions [1]. Traditionally, HAI surveillance constitutes a labour-intensive exercise, heavily dependent on manual data collection and the nuanced clinical insights of IPC specialists. The emergence of fully and semi-automated surveillance systems holds the promise of a significant turning point in IPC [13]. These novel systems aim to streamline data acquisition, improve analytical precision, and expedite intervention, thereby maximising the utilisation of human and financial resources. However, the successful deployment of these automated systems often depends on the availability of the required data in a structured, electronic form. Complicating this is the presence of multiple, sometimes discordant, IT solutions within healthcare settings. Despite these challenges, the transformative potential of automated systems to reshape traditional surveillance methodologies highlights the increasing role of information technology and data science in contemporary healthcare environments [14]. The PRAISE network, a collaboration involving 30 experts from 10 European countries, provides a comprehensive roadmap for transitioning from conventional manual surveillance to automated systems [15]. The guidance underscores the importance of uniform data, stakeholder engagement, and methodological re-evaluation as crucial steps for successful large-scale implementation to elevate the quality of care.

While automated surveillance offers considerable advantages, there is a noticeable gap in both the scholarly and practical discourse about its applicability to nvHAP. Given the condition's widespread prevalence and its implications for the health of virtually all hospitalised patients, it is imperative to assess the performance of automated surveillance systems in detecting nvHAP as a foundation for preventative measures. Additionally, the unique complexities and challenges associated with nvHAP, including surveillance definitions that typically rely on unstructured data formats for signs and symptoms, may necessitate tailored solutions distinct from those for other HAIs. A 2019 systematic review of electronically aided surveillance systems for HAIs in general also covered performance metrics for lower respiratory tract infections but did not distinguish between nvHAP and VAP [16]. In light of the rapidly evolving literature on automated nvHAP surveillance, our systematic review aims to fill this knowledge gap. We focus on delineating the current state of fully automated and semi-automated surveillance systems specific to HAP, with a special focus on nvHAP.

## Methods

We followed Preferred Reporting Items for Systematic Reviews and Meta-Analysis (PRISMA) recommendations when conducting this systematic review [17]. The study was registered at Prospero (Ref CRD42023444958). We searched Medline/Ovid, EMBASE, and the Cochrane Library for studies published before May 24th, 2023, without language restriction. The detailed search strategy was elaborated in collaboration with a health sciences librarian and is included in the Additional file 1. Duplicates were excluded, and additional articles were identified by reference list search from articles undergoing full-text review.

We incorporated studies that detailed automated surveillance methodologies for non-ventilated hospital-acquired pneumonia (nvHAP), as defined by the PRAISE Roadmap [15]. This encompassed both fully and semi-automated detection approaches, utilising data sources from electronic medical records, laboratory data and administrative claims data. Our review not only included articles specifically targeting nvHAP surveillance but also those focused on HAP overall, provided they did not exclusively concentrate on ventilator-associated pneumonia. We imposed no limitations on patient demographics or healthcare settings, embracing both hospital environments and other care facilities such as nursing homes or rehabilitation centres. The included articles were categorised based on whether they solely described the automated surveillance methodology or also provided validation of the system. Works limited to abstracts or posters were excluded from the review.

Two independent reviewers (AW and HS) conducted title and abstract screening. Any paper selected by either reviewer advanced to a full-text review stage. Subsequently, full-text evaluations were independently carried out by the same two reviewers. Discrepancies concerning article inclusion were deliberated between the two reviewers. In cases where consensus could not be reached, a third reviewer (AS) was consulted for final adjudication.

Utilising a standardised template, we extracted the following variables: year of publication, country, year and setting of surveillance, patient population, number of patients monitored, and the type of pneumonia (either nvHAP or HAP). We also catalogued the type of surveillance (fully or semi-automated), components incorporated into the selection algorithm, and incidence or incidence rates of nvHAP or HAP as determined by the automated surveillance. Additionally, the type of publication—whether it solely described the method or also included validation—was noted. For studies that validated their surveillance system, we further documented the type of reference standard used, and various performance metrics, including sensitivity, specificity, positive predictive value (PPV), negative predictive value (NPV), and workload reduction.

To evaluate the quality of the study design across all included papers, we employed the quality assessment instrument outlined by Streefkerk et al., utilising five of the six included quality indicators [16]. In the case of studies that validated automated surveillance methods against a reference standard, we used a modified version of the QUADAS-2 tool that was designed for assessing the quality of diagnostic accuracy studies, applying nine of its eleven ‘signalling questions’ [18].

Data were synthesised and presented in tables and within the full text. Given the considerable variability in automated surveillance methodologies, reference standards, and patient populations among the studies, we opted not to conduct a meta-analysis. Ethical approval was deemed unnecessary for this literature review.

## Results

**Fig. 1:**
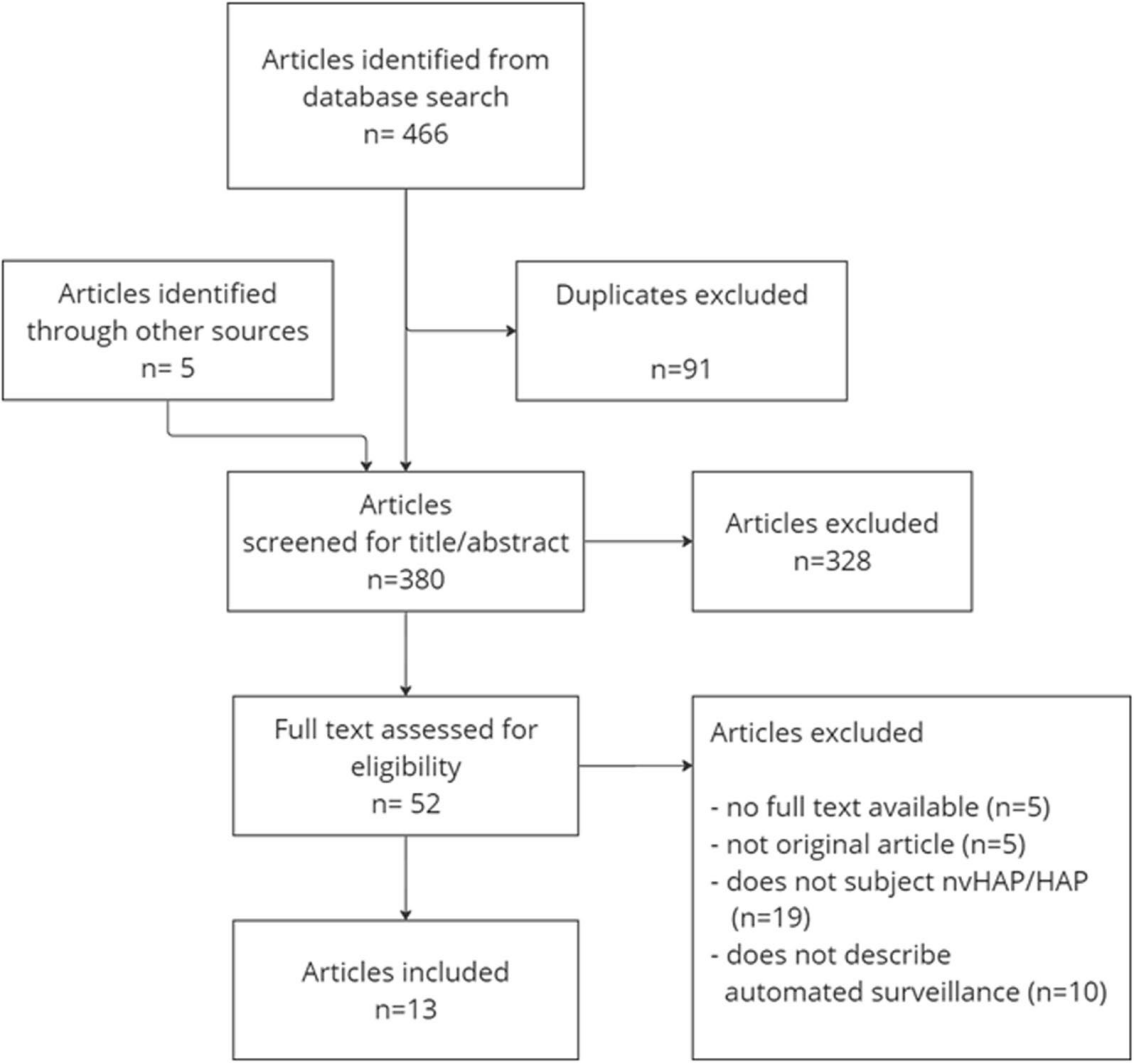
Study inclusion flow diagram

After eliminating duplicates, our database and manual reference searches yielded 380 articles. Following the screening of titles and abstracts, 328 articles were excluded, the full-text review of 52 articles left 13 (3.4% of the initial total) that satisfied our eligibility criteria and were included in the final review (Fig. 1). It is noteworthy that two of these articles described the same automated surveillance system and patient cohort, but each from a unique perspective—one from an infection prevention and control (IPC) [19], and the other from an information technology (IT) perspective [20]. These articles are jointly cited in subsequent sections [19, 20], bringing the count to 12 unique studies for our review.

Of the studies reviewed, 11 featured fully automated surveillance systems, while one showcased a semi-automated approach [21] (Table 1). Geographically, all the articles originated from high-income countries: eight from the United States, two from Switzerland, and one from Australia and France. All articles were published in 2005 or later, with nine (75%) appearing in or after 2018. Six studies specifically focused on non-ventilator hospital-acquired pneumonia (nvHAP), while the remaining six examined hospital-acquired pneumonia (HAP) more broadly.

**Table 1:** Overview of included studies

References	Type of pneumonia	Year of surveillance	Country	Setting	Patient cohort to whom surveillance was applied	Number of patients (admissions) the Surveillance was Applied on	Patient cohort used for validation	Number of patients included for validation	Number of described surveillance systems	Type of surveillance system (fully automatic vs. semi-automatic)	Validation	Number of reference methods applied
Stern [22]	nvHAP	2015–2020	USA	152 hospitals	Hospitalized adult patients	3.1 Mio (2.7 Mio^a^	Random selection of patients meeting automated surveillance criteria for nvHAP	250 (215^a^)	1	Fully	Yes	6
Jones [25]	nvHAP	2015–2020 and 2018- 2020	USA	284 hospitals	Hospitalised adult patients	6.0 Mio	Random selection of patients meeting automated surveillance criteria for nvHAP	250	1	Fully	Yes	1
Valentine [28]	HAP	2015–2017 and 2020- 2021	Austra-lia	1 hospital	Hospitalised patients with (hemato-)oncologic disease	41′260	All patients meeting automated surveillance criteria for nvHAP	151	1	Fully	Yes	1
Ramirez Battle [24]	nvHAP	2015–2017	USA	1 hospital	Random selection of patients with ≥ 3 day stay and with worsening oxygenation	120	Random selection of patients with ≥ 3 day stay and with worsening oxygenation	120	1	Fully	Yes	3
Lacerna [10]	nvHAP	2012–2018	USA	21 hospitals	Hospitalized patients	Not reported	n.a	n.a	1	Fully	No	n.a
Ji [23]	nvHAP	2015–2018	USA	4 hospitals	Hospitalized non-ventilated patients aged 18 years or older	489′519	Random selection of patients with ≥ 3 day stay and with worsening oxygenation	120	10	Fully	Yes	1
Zilberberg [30]	HAP (gramnegative)	2009–2016	USA	178 institutions	Adult patients with positive microbiology culture	Not reported	n.a	n.a	1	Fully	No	n.a
Wolfensberger [21]	nvHAP	2016–2017	Switzer-land	1 hospital	Hospitalized adult patients	39′519	3 samples: 1. Random sample of all patients; 2. Patients with discharge diagnostic code of HAP; 3. Random sample plus patients with discharge code of HAP	700 + 637 + 754 = 2091	1	Semi	Yes	1
Wolfensberger [27]	HAP	2016	Switzer-land	4 departments in 1 hospital	Hospitalized patients from 3 surgical and 1 medical department	6064	All patients meeting automated surveillance criteria plus random sample of patients who did not meet automated surveillance criteria	165 + 590 = 755	1	Fully	Yes	1
Fitzhenry [31]	HAP (postoperative pneumonia)	1999–2006	USA	6 hospitals	Patients with surgical procedures (inpatient and outpatient)	Not reported for patients with inpatient surgical procedure exclusively	Patients with inpatient surgical procedure with in-hospital postoperative pneumonia based on VASQIP surveillance	1017	1	Fully	Yes	1
Bouzbid [29]	HAP on ICU	2000–2006	France	1 department in 1 hospital	Patients from 1 ICU with hospital stay > 48 h	1499	Patients from 1 ICU with hospital stay > 48 h	1499	6	Fully	Yes	1
Haas [19] and Mendonca [20].	HAP on Neo	2001–2003	USA	1 department in 1 hospital	Hospitalized patients from a neonatal intensive care unit (NICU)	1688 or 1692^b^	Hospitalized patients from a neonatal intensive care unit (NICU)	1688 or 1692^b^	1	Fully	Yes	1

HAP, hospital-acquired pneumonia; ICU, intensive care unit; n.a., not applicable; Neo, neonatology; nvHAP, non-ventilator-associated hospital-acquired pneumonia; VASQIP, Veterans Affairs Surgical Quality Improvement Program

^a^Subset of patients for validation against discharge diagnostic codes

^b^Divergent numbers in papers of Haas et al. and Medonca et al

Table 2 delineates 24 unique candidate definitions for surveillance systems, 23 fully and 1 semi-automated, with each publication contributing between one and ten definitions. Four articles examined iterations of fully automated nvHAP surveillance systems that incorporate impaired oxygenation in various combinations including chest radiology, fever, leukocyte count, microbiology, and antibiotic use [22–25]. Chest radiology was included as an indicator in seven systems, leucocytosis or leukopenia in eight, and fever in nine. Two surveillance systems integrated radiology with fever or leucocytosis resp. leukopenia, aligning with the ECDC or (when coupled with altered mental status) the CDC's pneumonia definition criteria [1, 26]. Microbiology results were incorporated in nine systems, and antibiotic administration was part of 14. Three articles focused exclusively on automated surveillance systems using ICD-10 discharge diagnostic codes [27–29], while two others combined ICD-10 codes with additional algorithmic elements [10, 30]. Three studies explored surveillance systems that employed natural language processing of radiology reports, clinical notes, or discharge summaries [10, 19, 20, 31].

**Table 2:**
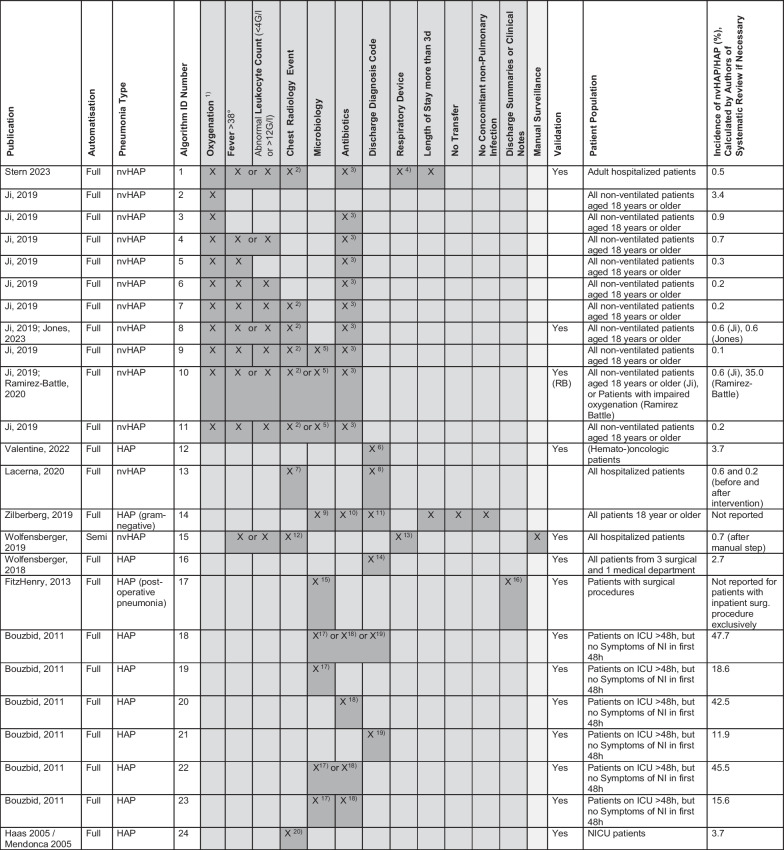
Algorithm components and incidence rates

^1)^Worsening oxygenation: defined as at least 2 days of stable or improving oxygenation followed by at least 2 days of (1) decrease in daily minimum oxygen saturation from at least 95% in a patient on ambient air to less than 95%on ambient air, (2) initiation of supplemental oxygen, or (3) escalation of supplemental oxygen. All additional criteria were required to be present on the first or second day of worsening oxygenation

^2)^Chest imaging obtained

^3)^Three days of new antibiotics (less than 3 days of new antibiotics was allowed if the patient died on the first or second day of antibiotics)

^4)^Non-intubated patients

^5)^Respiratory culture obtained

^6)^ICD-10-Australian Modification

^7)^Chest radiology including text analysis (a natural language processing searching imaging reports for opacity descriptors consistent with new pneumonia that persisted for > 24 h)

^8)^Discharge diagnosis of pneumonia occurring > 48 h after admission

^9)^Respiratory and/or blood culture specimen positive for at least one gram-negative organism obtained at hospital day 3 or later for HAP

^10)^Antibiotic treatment on day of respiratory culture and for a subsequent 3 days or more, or until death or discharge

^11)^ICD-9 pneumonia as a secondary (not primary) diagnosis

^12)^Radiological procedures with reports not containing key phrases ruling out pneumonia, and not performed within 48h after admission (unless re-admission

^13)^Permanent absence of respiratory device during 48h before radiology

^14)^ICD-10 U 69.00 proxy code for hospital-acquired pneumonia

^15)^Positive blood culture from microbiology report

^16)^General clinical notes or discharge summaries were parsed and mapped to SNOMED-CT concepts (“Lung consolidation” or “pneumonitis”) using a Natural Language Processing (NLP) program

^17)^Positive blood culture from microbiology report (NLP)

^18)^(1) antibiotic prescription (ATC: J01) and (2) antibiotic prescription 48 h after ICU admission or time antibiotic prescription changed ≥ 48 h after ICU admission

^19)^Primary or associated diagnosis of ICU stay coded by anatomical site (ICD 10) as follows: Pneumonia J10-, J11-, J12-, J13-, J14-, J15-, J16-, J17-, J18

^20)^Chest radiology reports including text analysis with an existing NLP system (MedLEE) and chest radiology not performed within 48h after admission

Among the 23 surveillance systems described, 14 underwent validation. Three algorithms (No. 1, 8, and 10) were validated against multiple reference standards [22–25], while one paper validated several algorithms (No. 18-23) against one single reference standard [29] (Table 3). Eight studies validated their automated systems using well-established criteria such as National Nosocomial Infections Surveillance System (NNIS) [19, 20], National Healthcare Safety Network—Centre for Disease Control and Prevention (NHSN-CDC) [22, 24, 25, 28], Hospitals in Europe Link for Infection Control through Surveillance/European Centre for Disease Prevention and Control (HELICS/ECDC) [21, 27, 29], or Veterans Affairs Surgical Quality Improvement Program (VASQIP) applied by manual chart review by one or two reviewers [31]. One publication described validation against discharge diagnostic codes [22], while two studies utilised diagnoses provided by treating physicians [22, 23]. Additional validations were performed against discharge summaries [22], nvHAP as defined by an expert reviewer [22, 24], or a composite of the aforementioned criteria [25].

**Table 3:** Performance characteristics of automated surveillance systems vs reference standards

References	Patient population	Number of patients	nvHAP/HAP	Algorithm ID number. ^a^	Reference type ID number^b^	Manual part of surveillance	Reference method	Sensitivity	Specificity	Positive predictive value	Negative predictive value
Stern [22]	Patients who met surveillance criteria	250	nvHAP	1	1		*CDC-NHS PNU1* both reviewers one reviewer	–	–	42% (39–46) 67% (64–70)	–
Stern [22]	Patients who met surveillance criteria	250	nvHAP	1	2		*Clinical nvHAP (treating physician)* both reviewers one reviewer	–	–	42% (39–46) 60% (57–63)	–
Stern [22]	Patients who met surveillance criteria	250	nvHAP	1	6		*Presence of clinical deterioration* both reviewers one reviewer	–	–	87% (85–89) 98% (98–99)	–
Stern [22]	Patients who met surveillance criteria	250	nvHAP	1	4		*Discharge summary nvHAP* both reviewers one reviewer	–	–	35% (32–38) 49% (46–52)	–
Stern [22]	Patients who met surveillance criteria	250	nvHAP	1	5		*Expert reviewer nvHAP* both reviewers one reviewer	–	–	50% (46–53) 71% (68–74)	–
Stern [22]	Patients who met surveillance criteria	250	nvHAP	1	7		VA claims based definition of nvHAP	–	–	7.9% (4–12)	–
Ji [23]	Patients with impaired oxygenation	120	nvHAP	8	2		Clinical nvHAP (treating physician)	56% (40–70)	77% (68–86)	60% (47–71)	–
Jones [25]	Patients who met surveillance criteria	250	nvHAP	8	2 or 4 or 5		Clinical nvHAP (treating physician) OR discharge summary nvHAP OR expert reviewer	–	–	81%	–
Ramirez-Batlle [24]	Patients with impaired oxygenation	120	nvHAP	10	1		CDC-NHSN PNEU	59% (39–77)	73% (62–81)	41% (30–52)	85% (78–90)
Ramirez-Batlle [24]	Patients with impaired oxygenation	120	nvHAP	10	2		Clinical nvHAP (treating physician)	56% (40–70)	77% (66–86)	60% (47–71)	74% (67–80
Ramirez-Batlle [24]	Patients with impaired oxygenation	120	nvHAP	10	3		“True pneumonia” (clinical plus AB-treatment)	71% (51–87)	76% (66–84)	48% (37–58)	90% (83–94)
Valentine [28]	Patients with hemato-oncolic disease	151	HAP post-EMR:34% pre-EMR:13%	12	1		CDC-NHSN PNU3	–	–	18% (12–25)	
Wolfensberger [21]	Random sample plus patients with ICD10	2091	nvHAP	15	1	x	ECDC nvHAP	98% (93–99) or higher	(100% not mentioned in the paper, but per definition)	(100% not mentioned in the paper, but per definition)	99% (98–100) or higher
Wolfensberger [27]	Patients from 3 surgical and 1 medical department	747 (590 random sample, 157 with ICD10)	HAP	16	1		HELICS/ECDC nvHAP	59% (48–69) (extrapolated)	98% (98–99) (extrapolated)	35% (27–42)	99% (98–100)
FitzHenry [31]	Procedures with surgical procedures	7743 patients with 8186 procedures (4098 in development and 4088 in validation cohort)	HAP (postoperative pneumonia)	17	1		VASQIP	79% (no CI)	–	–	–
Bouzbid [29]	Patients > 48 h on ICU	1499	HAP (on ICU)	18	1		HELICS	99% (98–100)	58% (56–61)	22% (19–25)	100% (99–100)
Bouzbid [29]	Patients > 48 h on ICU	1499	HAP (on ICU)	19	1		HELICS	86% (81–91)	89% (88–91)	49% (43–55)	98% (97–99)
Bouzbid [29]	Patients > 48 h on ICU	1499	HAP (on ICU)	20	1		HELICS	93% (89–97)	64% (61–66)	23% (20–27)	99% (98–99)
Bouzbid [29]	Patients > 48 h on ICU	1499	HAP (on ICU)	21	1		HELICS	40% (33–48)	92% (90–93)	36% (29–43)	93% (91–94)
Bouzbid [29]	Patients > 48 h on ICU	1499	HAP (on ICU)	22	1		HELICS	99% (97–100)	61% (58–63)	23% (20–26)	100% (99–100)
Bouzbid [29]	Patients > 48 h on ICU	1499	HAP (on ICU)	23	1		HELICS	81% (74–86)	92% (91–94)	55% (48–61)	98% (97–98)
Haas [19] and Mendonca [20]	NICU	1688 or 1692	HAP (on Neo)	24	1		NNIS-criteria	71% (no CI)	95% (no CI)	8% (no CI)	99.8% (no CI)

CDC-NHSN, Centre for Disease Control and Prevention—National Healthcare Safety Network; CI, confidence interval; ECDC, European Centre for Disease Prevention and Control; HAP, hospital-acquired pneumonia; HELICS, Hospitals in Europe Link for Infection Control through Surveillance; ICU, intensive care unit; ID, identification; NNIS, National Nosocomial Infections Surveillance; nvHAP, non-ventilator-associated hospital-acquired pneumonia; Neo, neonatology; PNEU, pneumonia; VASQIP, Veterans Affairs Surgical Quality Improvement Program

^a^Algorithm ID number: please refer to Table 2 to see the components of the specific algorithm

^b^Reference Type ID numbers: 1, well established surveillance definitions from CDC, ECDC, HELICS, NNIS or VASQIP; 2, clinical HAP diagnosed by treating physician; 3, “true pneumonia” based on clinical diagnosis and antibiotic treatment; 4, HAP according to discharge summary; 5, HAP according to expert reviewer; 6, presence of clinical deterioration; 7, claims based HAP

For fully automated surveillance, the sensitivity of the algorithms varied between 40 and 99%, specificity ranged from 58 to 98%, PPV spanned from 8 to 71%, and NPV extended from 74 to 100%. The only study describing semi-automated surveillance reported a sensitivity and NPV of 98% and 99%, respectively [21]. While all fully automated surveillance systems inherently achieve a 100% reduction in workload, the actual time saving was not reported in any of the studies. The only semi-automated system documented a 94% decrease in patients requiring manual screening but did not report the time reduction either [21].

Table 4 presents the quality scores for the included papers, which varied from 10 to 23 out of a possible 25 points as per the modified quality assessment tool by Streefkerk et al. [16]. Suboptimal scoring was common in separating the test from the validation cohorts (“Indicator 1”), as only one study included a separate derivation and a validation cohort [31], and in reporting the scope of performance characteristics (“Indicator 5”) with five studies scoring 0 because they did not validate the automated system or did not report sensitivity. The scores achieved in the adapted QUADAS-2 instrument [18], ranged between 7 and 9 out of a maximum of 9 points. Seven studies scored 0 in either the item “Did the study avoid inappropriate exclusion?” or “Did all patients receive a reference standard?” as either the surveillance or the reference standard was only applied to a subset of patients.

**Table 4:** Quality rating of studies

References	Quality of study designs according Streefkerk et al							Quality assessment according to QUADAS-2									
	Indicator 1: Validation and test cohort (5 points), test cohort only (3 points), validation cohort only (1 point)	Indicator 2: Prevalence or incidence of nvHAP/HAP reported (5 points), not reported (1 point)	Indicator 3: Hospital-wide surveillance for nvHAP/HAP (5 points), surveillance in > 2 departments or wards (3 points), surveillance in 1 ward/department (1 point)	Indicator 4: Types of HAI under surveillance → not applicable	Indicator 5: Sensitivity, specificity and other performance measure reported (5points), sensitivity and specificity reported (3 points), sensitivity reported (1 point), no or other measure than sensitivity reported (0 points)	Indicator 6: time reduction was presented quantitatively (5 points), workload reduction was presented (3 points), no data on workload reduction presented (1 point)	Sum of Streefkerk et al. (Max = 25)	Question 1. Was a consecutive or random sample of patients enrolled?	Question 2. Was a case–control design avoided?	Question 3. Did the study avoid inappropriate exclusions?	Question 4. Were the index test results interpreted without knowledge of the results of the reference standard?	Question 5. Is the reference standard likely to correctly classify the target condition?	Question 6. Were the reference standard results interpreted without knowledge of the results of the index test?	Question 7. Did all patients receive a reference standard?	Question 8. Did all patients receive the same reference standard?	Question 9. Were all patients included in the analysis?	Sum of QUADAS-2 (Max = 9)
Stern [22]	1	5	5	n.a	0	3^a^	14	1	1	1	1	1	0	0	1	1	7
Jones [25]	1	5	5	n.a	0	3^a^	14	1	1	1	1	1	0	0	1	1	7
Valentine [28]	1	5	5	n.a	0	3^a^	14	1	1	1	1	1	0	0	1	1	7
Ramirez Battle [24]	1	5	5	n.a	5	3^a^	19	1	1	0^b^	1	1	1	1^c^	1	1	8
Lacerna [10]	1	5	5	n.a	0	3^a^	14	–	–	–	–	–	–	–	–	–	–
Ji [23]	1	5	5	n.a	5	3^a^	19	1	1	1	1	1^d^	1	0	1	1	8
Zilberberg [30]	1	1	5	n.a	0	3^a^	10	–	–	–	–	–	–	–	–	–	–
Wolfensberger [21]	1	5	5	n.a	1	3	15	1	1	1	1	1	1	0	1	1	8
Wolfensberger [27]	1	5	3	n.a	5	3^a^	17	1	1	1	1	1	0	0	1	1	7
Fitzhenry [31]	5	5	5	n.a	5	3^a^	23	1	0^e^	1	1	1	1	0	1	1	7
Bouzbid [29]	1	5	1	n.a	5	3^a^	15	1	1	1	1	1	1	1^f^	1	1	8
Haas and Mendonca [19]	1	5	1	n.a	5	3^a^	15	1	1	1	1	1	1	1	1	1	9

HAI, healthcare–associated infection

^a^Automated surveillance, 100% workload reduction assumed

^b^Only patients with worsening oxygenation

^c^Of the preselected patients undergoing surveillance, see Quadas-2 Question 3

^d^Clinically diagnosed nvHAP

^e^Patients with complication plus a 10% random sample

^f^Patients with stay > 48 h

## Discussion

We performed a systematic literature review on automated surveillance of HAP, with a specific focus on nvHAP. We found 13 articles representing 12 distinct studies, with 9 published after 2018, of which 6 focussing specifically on nvHAP [10, 21–25]. Except for one article, all described fully automated systems, featuring 24 different candidate definitions for surveillance. Validation was performed for 14 of these systems and relied on a range of mostly manual reference standards, most frequently employing definitions from authoritative organisations like the ECDC and the CDC. The performance of the fully automated surveillance systems varied, with higher sensitivity often correlated with lower positive predictive values (PPV) and vice versa.

Key metrics for evaluating automated surveillance systems include sensitivity, specificity, PPV, and NPV. The PRAISE network emphasises the importance of these metrics and recommends study designs to minimise differential and partial bias [15]. In our review, all but one validation study reported PPV. The majority also reported sensitivity, specificity, and NPV. The one semi-automated system we reviewed stood out with a sensitivity of 98% [21]. According to guidelines by van Mourik et al., semi-automated systems should ideally achieve a sensitivity above 90% [15]. In contrast, the fully automated systems demonstrating high sensitivity, often lagged in PPV and specificity. Such inconsistencies in wrongly classifying patients as having nvHAP could undermine trust among clinicians and administrators. Yet, Stern et al. point out that manual surveillance is not without its own reliability issues, the authors found a simple agreement between two reviewers assessing patients for CDC-NHSN pneumonia criteria of 75% and a moderate interrater agreement (Cohen Kappa: 0.5) [22]. This suggests that automated systems offer reliability comparable to human operatives. While the subjectivity, complexity, and ambiguity of clinical and surveillance definitions for pneumonia have been extensively debated [22], the gold standard for diagnosis, namely pathology, is seldom available. Currently, there are no universally accepted guidelines for validating automated HAI surveillance systems, leaving key questions about the minimal number of reviewers and performance criteria unanswered. Establishing such guidelines would significantly advance the development and validation of automated systems for nvHAP and other HAIs. Streefkerk et al. suggested an overall performance score (i.e. multiplying sensitivity and specificity) of ≥ 0.85 as a standard [16]. Notably, none of the fully automated systems in our review met this criterion.

Most validation studies in this review, except for two [19, 20, 29], assessed automated systems on preselected patient groups. Such selection often limits the system's applicability to a broader patient base. Furthermore, many studies had small sample sizes, between 120 and 250 patients, leading to less precise performance metrics.

Broadly, the identified automated surveillance systems fall into three categories: those utilising clinical data (some applying NLP methods for data extraction), those relying on discharge diagnostic codes, and those employing a combination of both). Systems relying mainly on pneumonia discharge codes show poor results, with sensitivities between 40 and 60% and even lower PPVs of 18–36% [27–29], raising questions about their inclusion in algorithms. In terms of components of systems using clinical data, earlier studies used factors like microbiology and antibiotic prescriptions, while recent ones focus on internationally accepted nvHAP criteria [1, 26], such as radiology, fever, and abnormal leukocyte counts. Antibiotic use is frequently included, given its role in treating HAP, which are rarely of viral aetiology only [21]. A group of researchers has significantly shaped this field since 2019, developing automated systems based on CDC definitions for pneumonia and ventilator-associated events [32, 33]. These systems focus on "worsening oxygenation" as a key criterion [22–25], and have been tested across multiple hospitals in pre-selected patient groups with deteriorating oxygen levels. Depending on the manual reference method and the candidate surveillance definition, sensitivities ranged from 56 to 71% and PPVs from 35 to 81%. However, the focus on deteriorating oxygen levels is debatable. While such patients may be more likely to experience adverse outcomes like ICU admission and death, the extent of nvHAP occurrence among patients who do not experience oxygenation impairment is still unknown. Considering antibiotic stewardship, this latter group could also significantly impact the number of preventable antibiotic prescriptions.

While currently many existing surveillance systems rely on structured data formats, established definitions and clinical diagnoses of pneumonia often include symptoms or findings typically recorded in unstructured text, such as clinical notes or discharge summaries, or images. Although three studies applied natural language processing (NLP) technology, the potential of artificial intelligence (AI) was not yet fully exploited in the published studies. The inclusion of AI could address this gap and further limiting manual work in semi-automated surveillance or increasing the performance of fully automated surveillance. Initial efforts date back to as early as 2005, spearheaded by researchers like Mendonca and Haas et al. [19, 20]. These advancements show great potential for incorporating often-overlooked symptomatology, such as coughing or auscultation findings, into future automated surveillance systems. For example, cutting-edge technologies like GPT-4, as explored by Perret and Schmid [34], could facilitate such integration. Furthermore, AI algorithms have already demonstrated capabilities that equal or surpass radiologists in identifying singular anomalies in chest X-rays [35].

Our review has limitations. While we aimed to include all validation studies on automated nvHAP surveillance, we may have missed some without validation that were part of intervention studies. The studies we did include showed considerable heterogeneity in study methodologies, surveillance algorithms, patient cohorts, and quality indicators, making a meta-analysis to calculate a collective performance impractical and prohibited a precise identification of most promising system elements. The lack of multi-setting validation and the small sample sizes in most studies affect our conclusions' robustness [21–23, 27, 28, 31].

## Conclusion

Automated surveillance undeniably reduces workload, allows real-time reporting, and enables rapid interventions. Progress has been made in recent years to develop and validate automated nvHAP surveillance systems. However, the varied study designs and validation methods reviewed do not allow us to conclusively determine which features of nvHAP surveillance algorithms are most effective. From a standpoint of careful analysis and practical insights, some general advice can be offered. Firstly, we recommend to integrate indicators in nvHAP selection algorithms that are universally present in all nvHAP patients, such as radiology. For indicators with lower sensitivity, such as discharge diagnostic codes or positive microbiology results, a judicious application is advised. These might still be used as optional criteria or components of a sophisticated multivariable regression model. When the sensitivity of a specific indicator is uncertain, a detailed evaluation in a larger patient cohort with confirmed (nv)HAP, determined through manual surveillance, is essential. Incorporating recognised surveillance elements like fever or abnormal leucocyte counts can enhance the alignment with manual methods. Although the end goal is a fully automated HAP surveillance system, adopting semi-automated systems in the interim might be a practical approach, at least until the reliability of fully automated systems is indisputably established. Currently, the adequacy of fully automated systems, as indicated by the available performance metrics, remains a subject for debate. To provide a more conclusive evaluation, future research should employ a rigorous validation process to avoid bias and include broad patient populations. The implementation of emerging AI techniques holds the potential to revolutionise surveillance in the near future, provided challenges such as data privacy and AI biases can be overcome [36]. The capability of AI to mine extensive information from unstructured clinical data, especially concerning symptomatology and radiology, could significantly enhance the performance of automated surveillance systems..

### Supplementary Information

**Additional file 1**. Embase Search Strategy.

## Data Availability

Not applicable.
