# Supplementary material for: Automated surveillance of non-ventilator-associated hospital-acquired pneumonia (nvHAP): a systematic literature review
Source: Antimicrob Resist Infect Control. 2024 Mar 6;13:30. doi: 10.1186/s13756-024-01375-8 (PMC10918924; doi:10.1186/s13756-024-01375-8)
Supplement: Supplementary file 1 — Additional file 1. Embase Search Strategy. [file 13756_2024_1375_MOESM1_ESM.pdf]

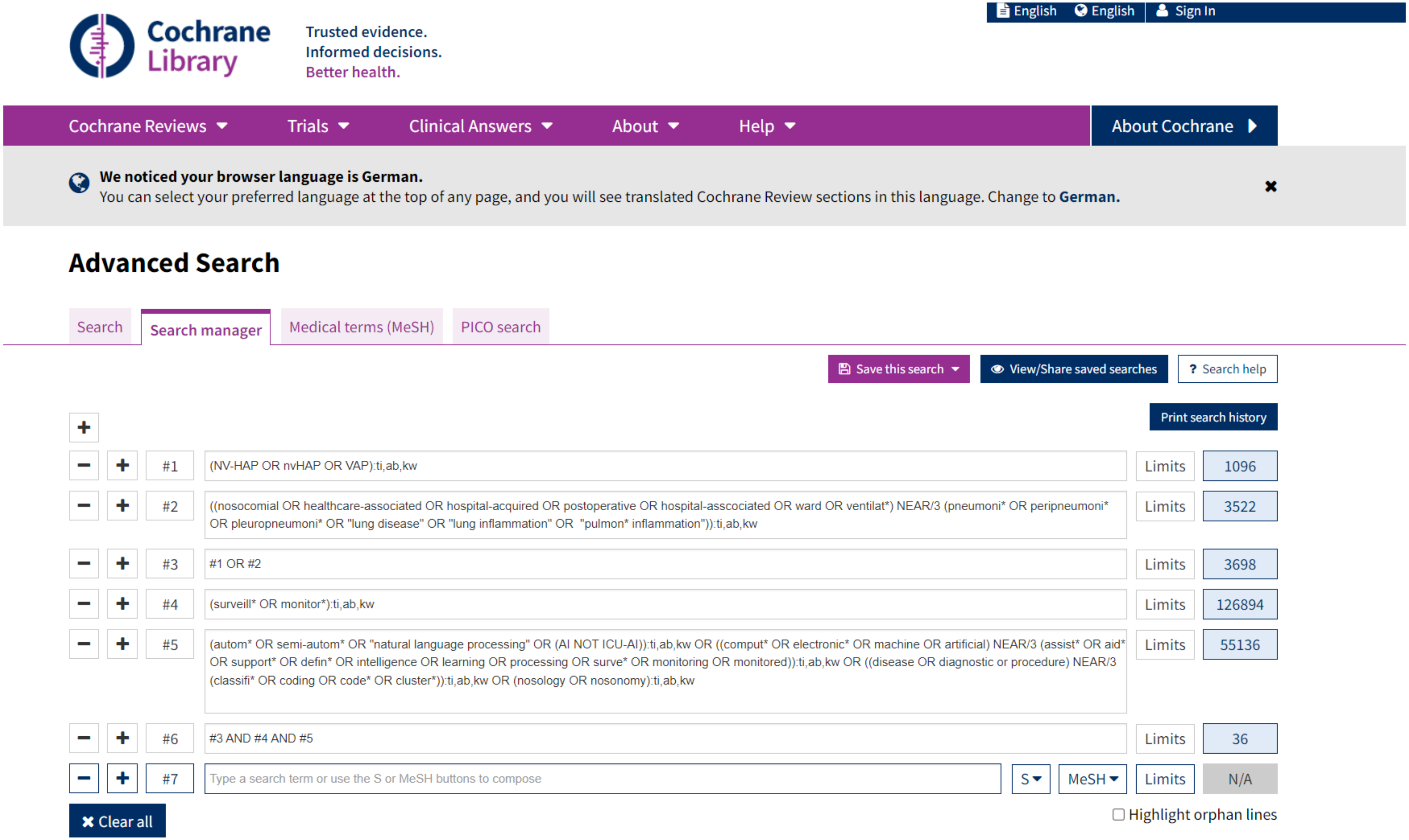

Caption: Cochrane Library Search Strategy

Embase

Embase session results (24 May 2023)

No.	Query	Results
#9	#7 OR #8	302
#8	#1 AND #2 AND #3 NOT (('animal'/de OR 'animal experiment'/exp OR 'nonhuman'/de) NOT ('human'/exp OR 'human experiment'/de)) NOT [conference abstract]/lim	282
#7	#1 AND #2 AND #3 NOT (('animal'/de OR 'animal experiment'/exp OR 'nonhuman'/de) NOT ('human'/exp OR 'human experiment'/de)) AND [conference abstract]/lim AND [2020-2023]/py	20
#6	#1 AND #2 AND #3 NOT (('animal'/de OR 'animal experiment'/exp OR 'nonhuman'/de) NOT ('human'/exp OR 'human experiment'/de)) AND [conference abstract]/lim	193
#5	#1 AND #2 AND #3 NOT (('animal'/de OR 'animal experiment'/exp OR 'nonhuman'/de) NOT ('human'/exp OR 'human experiment'/de))	475
#4	#1 AND #2 AND #3	488
#3	'information processing'/exp OR 'artificial intelligence'/exp OR 'natural language processing'/exp OR 'automation'/exp OR 'disease classification'/exp OR 'international classification of diseases'/exp OR autom*:ti,ab,kw OR 'semi autom*':ti,ab,kw OR 'natural language processing':ti,ab,kw OR (ai:ti,ab,kw NOT 'icu ai':ti,ab,kw) OR (((comput* OR electronic* OR machine OR artificial) NEAR/3 (assist* OR aid* OR support* OR defin* OR intelligence OR learning OR processing OR surve* OR monitoring OR monitored)):ti,ab,kw) OR (((disease OR diagnostic OR procedure) NEAR/3 (classifi* OR coding OR code* OR cluster*)):ti,ab,kw) OR nosology:ti,ab,kw OR nosonomy:ti,ab,kw	3804198
#2	'monitoring'/de OR 'disease surveillance'/exp OR surveill*:ti,ab,kw OR monitor*:ti,ab,kw	1676741

#1	'hospital infection'/exp AND 'pneumonia'/exp OR 'hospital acquired pneumonia'/exp OR 'nv hap':ti,ab,kw OR nvhap:ti,ab,kw OR vap:ti,ab,kw OR (((nosocomial OR 'healthcare associated' OR 'hospital acquired' OR postoperative OR 'hospital asscociated' OR ward OR ventilat*) NEAR/3 (pneumoni* OR peripneumoni* OR pleuropneumoni* OR 'lung disease' OR 'lung inflammation' OR 'pulmon* inflammation')):ti,ab,kw)	32001

Copyright © 2023 Elsevier Limited except certain content provided by third parties.

Embase is a trade mark of Elsevier Life Sciences IP Limited.

Caption: Embase Search Strategy

Ovid®
Wolters Kluwer
Support & Training Feedback Close

Database(s): **Ovid MEDLINE(R) and Epub Ahead of Print, In-Process, In-Data-Review & Other Non-Indexed Citations and Daily** 1946 to May 23, 2023
Search Strategy:

#	Searches	Results
1	(exp cross infection/ and exp pneumonia/) or exp healthcare-associated pneumonia/ or (NV-HAP or nvHAP or VAP).ti,ab,kf. or ((nosocomial or healthcare-associated or hospital-acquired or postoperative or hospital-asscociated or ward or ventilat*) adj3 (pneumoni* or peripneumoni* or pleuropneumoni* or "lung disease" or "lung inflammation" or "pulmon* inflammation")).ti,ab,kf.	19386
2	exp Population Surveillance/ or (surveill* or monitor*).ti,ab,kf.	1232569
3	exp Electronic Data Processing/ or exp computing methodologies/ or exp Artificial Intelligence/ or exp Natural Language Processing/ or exp Automation/ or exp "International Classification of Diseases"/ or (autom* or semi-autom* or "natural language processing" or (AI not ICU-AI)).ti,ab,kf. or ((comput* or electronic* or machine or artificial) adj3 (assist* or aid* or support* or defin* or intelligence or learning or processing or surve* or monitoring or monitored)).ti,ab,kf. or ((disease or diagnostic or procedure) adj3 (classifi* or coding or code* or cluster*)).ti,ab,kf. or (nosology or nosonomy).ti,ab,kf.	1605071
4	1 and 2 and 3	132
5	(1 and 2 and 3) not (animals not humans).sh.	128

1. **Electronic surveillance** criteria for non-ventilator-associated **hospital-acquired pneumonia**: Assessment of reliability and validity.

   Stern SE, Christensen MA, Nevers MR, Ying J, McKenna C, Munro S, Rhee C, Samore MH, Klompas M, Jones BE

   Infection Control & Hospital Epidemiology. 1-7, 2023 Mar 15.

   [Journal Article]

   UI: 36920040

   Authors Full Name
   Stern, Sarah E, Christensen, Matthew A, Nevers, McKenna R, Ying, Jian, McKenna, Caroline, Munro, Shannon, Rhee, Chanu, Samore, Matthew H, Klompas, Michael, Jones, Barbara E

   Cite

Caption: Medline/Ovid Search Strategy
